# Assessment of financial toxicity in patients with cancer in Slovenia

**DOI:** 10.1007/s00520-025-09591-7

**Published:** 2025-05-30

**Authors:** Katja Vöröš, Marjeta Skubic, Mojca Bavdaž, Petra Došenović Bonča, Andraž Perhavec, Tjaša Redek, Helena Barbara Zobec Logar, Ivica Ratoša

**Affiliations:** 1 Faculty of Medicine, University of Ljubljana, Ljubljana, Slovenia; 2 School of Economics and Business, University of Ljubljana, Ljubljana, Slovenia; 3 Division of Surgical Oncology, Institute of Oncology Ljubljana, Ljubljana, Slovenia; 4 Division of Radiotherapy, Institute of Oncology Ljubljana, Zaloska Cesta 2, 1000 Ljubljana, Slovenia

**Keywords:** Financial toxicity, Cancer, Questionnaire, Finances, Financial burden, Out-of-pocket expenditures

## Abstract

**Purpose:**

The aim of this study was to assess the extent of financial toxicity in patients with cancer in Slovenia, measured as perceived financial strain and out-of-pocket expenditures.

**Methods:**

The prospective, cross-sectional study was done at Institute of Oncology Ljubljana from June to October 2023. A newly created individual questionnaire was utilized to obtain data on financial toxicity. Patients completed the questionnaire either on paper or online, with or without the assistance of a researcher. The statistical analysis was based on descriptive and inferential statistics.

**Results:**

A total of 901 surveys were disseminated, of which 659 were returned, corresponding to a response rate of 73%. Most patients had compulsory health insurance and were diagnosed in the public healthcare system. Following a cancer diagnosis, 178 (27%) patients reported a decline in financial satisfaction (subjective assessment), while 150 (22.7%) patients observed a change in their financial capability (objective assessment). Younger patients (*p* < 0.001), female patients (*p* < 0.004), patients with lower net household income (*p* < 0.001), and patients who were employed before the diagnosis (*p* < 0.001) were at a greater risk for financial toxicity. Patients with breast cancer and colorectal, endometrial, skin (including melanoma), esophageal, stomach, kidney, bladder cancers, and lymphoma had higher self-reported subjective and objective financial toxicity than patients with prostate, lung, and head and neck cancer.

**Conclusion:**

This article represents the first comprehensive assessment of financial toxicity among patients with cancer in Slovenia, using perceived financial strain and out-of-pocket expenditures. Most patients reported no significant out-of-pocket costs for the treatment they received. About a quarter of patients with cancer experience financial difficulties.

**Supplementary Information:**

The online version contains supplementary material available at 10.1007/s00520-025-09591-7.

## Introduction

Financial toxicity (FT) is a term used to describe the subjective and objective financial distress caused by cancer, which have a major impact not only on patients but also on society as a whole [1]. The term was first used in 2013 by Zafar et al. while studying the FT of insured patients in the USA [2]. The escalation of cancer-related expenses can be attributed to four primary factors: an aging population, more patients with access to treatment, advancements in medical innovation, and overutilization of resources. Out-of-pocket treatment expenses resemble physical toxicity, since they can reduce quality of life and hinder the provision of optimal care. The escalating expenses of cancer treatment are mostly addressed within health policy discussions, focusing on the financial implications for society. Current studies have indicated both objective financial burden and subjective financial hardship as critical elements of financial toxicity [2]. Many studies have shown that high FT is associated with treatment attrition, poor quality of life, and poor prognosis [3].

Cancer is far more common in the elderly population. World population data indicate that in 2020, the estimated incidence of cancer cases in the 0–64 age group was about 9.344 million new cases and in the ≥ 65 age group 9.949 million new cases, while the ≥ 65 age group represented less than a tenth of the world population [4]. Similar trends can be observed in Slovenia, where patients older than 65 account for two thirds (65%) of all new cases while accounting for a fifth of the population [5]. A growing number of people worldwide are being diagnosed with cancer each year, which comes with a growing financial burden [6].

A balance of public and private financing is present in the universal health systems of numerous countries that have been assessed by the Organisation for Economic Co-operation and Development (OECD). However, the manner in which each country manages this balance varies, as the fraction paid privately varies from country to country depending on historic, social, and economic factors. This implies that the health services that patients are required to pay for out-of-pocket and those that are “free” or covered by public financing differ from one country to another [7].

Slovenia’s health care system is founded on the Bismarck model, featuring compulsory health insurance administered by a singular state insurer, namely the Health Insurance Institute of Slovenia. The latter guarantees the provision of a comprehensive array of publicly supported health services, as delineated in the Act on Health Care and Health Insurance. Compulsory health insurance entitles individuals to services at primary, secondary, and tertiary levels, medications, medical devices, financial compensation for sickness absence exceeding 30 days, and, in certain instances, reimbursement of transport expenses to medical facilities. Mandatory health insurance offers comprehensive coverage for many services, encompassing emergency medical care, infectious diseases, family planning, hospital care, and social welfare facilities, as well as treatment and rehabilitation for malignant diseases [8].

Several factors contribute to FT of cancer, including socio-demographic characteristics such as gender, age, educational level, religion, and location (urban/rural). The number of regularly employed household members, household type, income level, and compulsory health insurance coverage also play a role in determining the socioeconomic status. A reduced FT is observed among members with higher income and more consistent employment [9]. An important set of variables includes characteristics of the disease, treatment, and healthcare system [10]. Some cancers are associated with higher FT, e.g., head and neck cancer, colorectal cancer, and hematologic cancers, since their treatment takes longer or consists of expensive therapies [11, 12]. Furthermore, the type of cancer treatment contributes to FT in different ways, e.g., multimodal treatment is associated with higher costs due to usually longer sick leave, higher travel costs to the health facility because of more visits, and a greater need for additional medical equipment, medicines or nutritional supplements [13]. FT may also be due to costs, such as medical equipment, e.g., wheelchairs, crutches, wigs, breast prostheses, over-the-counter (OTC) medicines, nutritional supplements, transport costs, parking fees, food and lodging, home adaptations, health and personal care assistance, physiotherapy, and psychological treatment [14]. Loss of income caused by sick leave, reduced working hours or disability retirement is also an important factor [15]. In addition, some patients seek alternative therapies, e.g., cannabis, bio-resonance, or traditional healers [16].

Most tools evaluating financial distress were designed for countries with private healthcare systems. One of the most widely used tool for measuring FT in cancer patients is the Comprehensive Score for Financial Toxicity-Functional Assessment of Chronic Illness Therapy (COST-FACIT) [17]. However, patients with cancer in countries with private healthcare experience higher FT, as health services and medicines are usually paid for in part or in full out of patients’ own pockets, even if they have insurance. It is therefore not surprising that the prevalence of FT in the USA is 39–64% [18], compared to 22–27% in countries with public healthcare systems [19]. In Slovenia, 99% of citizens have compulsory health insurance, which covers most of the healthcare services, including urgent medical treatment and all treatments for patients with cancer, treated according to national and international guidelines [20].

Despite the existence of established tools, including the PROFFIT measure (16-item Patient-Reported Outcome for Fighting Financial Toxicity) [21] and the 9-item Financial Index of Toxicity questionnaire, which assess financial stress, financial strain, and lost productivity [22], we chose to create a tailored questionnaire to assess the level of financial capabilities and satisfaction with personal finances among cancer patients in Slovenia. Furthermore, we quantified the costs incurred directly by individuals.

## Material and methods

The study was approved by the Institutional Review Board and Ethics Committee (approval number ERIDNPVO-0031/2023) and by the National Medical Ethics Committee (approval number 0120–105/2023/3). The study was conducted in accordance with the Declaration of Helsinki. All patients signed an informed consent form to participate in the study and allow the processing of their personal data. A prospective cross-sectional study at the Institute of Oncology Ljubljana was conducted from June 2023 to October 2023.

### Patients

The selection of patients with cancer was based on the following inclusion criteria: they had to be at least 18 years old, have had a confirmed cancer diagnosis, and provided informed consent to participate in the study. Having cognitive impairment that prevented them from completing the questionnaire was an exclusion criterion. Patients answered the questionnaire on their own or with the assistance of trained researchers. Participants had the option to either fill out the questionnaire manually on paper or utilize the online application OneClickSurvey [23]. Chart reviews were used to obtain patient information.

### Questionnaire development

To identify factors contributing to financial toxicity, we conducted a comprehensive literature search using PubMed, Google Scholar, and Web of Science to identify peer-reviewed original research articles, meta-analyses, and reviews published until February 2024. Search details and PRISMA flow diagram are presented in the online supplementary information (Supplementary information 1). Following an extensive literature review and analysis of existing instruments for assessing FT, we designed a tailored questionnaire for the publicly funded healthcare system in Slovenia. In the initial phase, 50 patients participated in face-to-face (F2 F) interviews to test the questionnaire through pilot testing. Following our assessment of the data and feedback, we took note of the patients’ comments on the questionnaire and identified areas for improvement. Experts in methodology, statistics, economics, and oncology also evaluated the questionnaire. The questionnaire was divided into four sections: disease and treatment, socioeconomic situation, focused questions on financial capacity, and sociodemographic data, comprising in total 38 questions. Supplementary material provides further information about the questionnaire’s development and the topics included (Supplementary Information 1). The subjective part of the questionnaire (subjective FT) included patients’ contentment with their financial circumstances, potential concerns over their financial status, and comparisons with the financial situations of other families. Objective questions (objective FT) focused on the ability to pay for food, medicine, and home bills each month. Most survey questions were multiple choice questions with either single-answer or multiple-answer format. Other questions asked for a number or free text (Supplementary Information 2).

### Statistics

We used Microsoft® Excel® for Office 365, version 1812 (Microsoft Corporation, One Microsoft Way Redmond, WA, USA) to process the data and prepare the figures. For further statistics and analysis, we used IBM® SPSS®, version 29.0.0.0. (Statistical Package for the Social Sciences Statistical Software; SPSS inc, Armonk: NY, IBM Corporation). Data were summarized using frequencies and percentages. Means and standard deviations were calculated. Where indicated, we used the median with a range. Kolmogorov–Smirnov and Shapiro–Wilk tests were used to test the normality of the data. Due to the non-normal distribution, we used the non-parametric Kruskal–Wallis test to analyze the variation. The distribution of specific parameters between the observed patient subgroups was compared using the chi-square test. The results were considered statistically significant if a two-sided *p*-value was below 0.05.

## Results

A total of 901 surveys were disseminated, of which 659 were returned, corresponding to a response rate of 73%. Our analysis was limited to 587 (65%) completed questionnaires. A total of 571 (97.2%) patients completed the questionnaire in paper format, while 16 (2.7%) patients completed it online. The median age of the participants was 64 years (range, 25–90). All other patient demographics, primary cancer diagnosis, and treatment are shown in Table 1.

**Table 1:** The patients’ demographic data, primary cancer diagnosis, and treatment

Parameter	Number *N* = 587	Percentage (%) 100%
Age		
(≤ 65)	324	55.2
(> 65)	263	44.8
Gender		
Male	291	49.6
Female	293	49.9
NA	3	0.5
Area of living		
Urban	283	48.2
Rural	298	50.8
NA	6	1.0
Level of education		
Levels 1 and 2	81	13.8
Levels 3 and 4	320	54.5
Levels 5–8	181	30.8
NA	5	0.9
Religion		
Christianity	392	66.7
Judaism	1	0.2
Islam	13	2.2
Atheism	129	22.0
Other	15	2.6
NA	37	6.3
Primary cancer diagnosis		
Breast cancer	118	20.1
Prostate cancer	111	18.9
Lung cancer	97	16.5
Head and neck cancer	66	11.3
Colorectal cancer	45	7.7
Endometrial cancer	40	6.8
Lymphoma	33	5.6
Skin cancer (including melanoma)	24	4.1
Esophageal and stomach cancer	12	2.0
Kidney cancer	6	1.0
Bladder cancer	4	0.7
Other	31	5.3
Treatment characteristics		
Multimodality treatment*	421	71.7
Radiation therapy (only)	77	13.1
Chemotherapy (only)	48	8.2
Surgery (only)	26	4.4
Endocrine therapy (only)	8	1.4
Targeted therapy (only)	5	0.9
NA	2	0.3

*Includes surgery, systemic treatment (any), or radiation therapy. *NA* not available/no data. Levels of education: [1] incomplete primary education, [2] primary education, [3] lower or secondary vocational education, [4] secondary vocational, general education, [5] higher education, post-secondary education, [6] higher vocational education, [7] university higher education qualification, [8] specialization, Master of Science, Doctorate

The majority of participating patients were diagnosed within the public healthcare system due to symptoms and signs (*n* = 431, 73.4%), in screening programs (*n* = 55, 9.4%), preventive screening (*n* = 65, 11.1%), or for other reasons (*n* = 3, 0.1%), with only a tenth diagnosed in the free-market healthcare (*n* = 60, 10.2%). The total is larger than 100% because several answers were possible to this question. The majority (*n* = 493, 84%) of patients were diagnosed with cancer for the first time, while 94 (16%) patients were diagnosed with cancer recurrence. Most patients in the study were undergoing active therapy (*n* = 406, 69.2%), followed by patients in follow-up after primary cancer treatment (*n* = 118, 20.1%), patients on a “watch and wait” approach (*n* = 48, 8.2%), and patients in palliative care (*n* = 14, 2.4%). We had no information on treatment activity for one patient. The socio-economic status of patients is shown in Table 2.

**Table 2:** Socio-economic status of the patients

Type of household	Number *N* = 587	Percentage (%) 100%
Live alone	80	13.7
Live with one or more other people/family members	501	85.3
Live in a nursing home	4	0.7
Other	2	0.3
Number of people living in the same household		
1 person	80	13.7
2 people	268	45.7
3 people	99	16.9
4 people	76	12.9
5 people or more	52	8.9
NA	12	2.0
Type of health insurance*		
Compulsory health insurance	585	99.7
Complementary health insurance**	585	99.7
Supplementary health insurance***	36	6.1
Health insurance from abroad	1	0.2
Temporary protection for displaced persons	1	0.2
Net income per family member		
> 1500 EUR	65	11.1
1200–1500 EUR	80	13.6
900–1200 EUR	108	18.3
600–900 EUR	207	35.3
300–600 EUR	105	17.9
< 300 EUR	18	3.1
NA	4	0.7

*NA* not available/no data

*Multiple answers were possible

**Complementary health insurance covers the difference between total price of the service and share of the price of this service covered by compulsory health insurance

***Supplementary health insurance covers costs of health care services that are not covered by compulsory health insurance, complementary health insurance or substitutive health insurance

No significant differences in income levels were found between the five main groups of primary cancer diagnosis (breast cancer, prostate cancer, lung cancer, head and neck cancer, and “all other cancers”; *p* = 0.104), age (≤ 65 years versus > 65 years; *p* = 0.053), or gender (*p* = 0.390).

Before the onset of the disease, around half of the patients (*n* = 299, 50.9%) were retired or retired with full disability. Others reported being self-employed or working full-time (*n* = 242, 41.2%) or part-time (*n* = 14, 2.4%). Some were unemployed (*n* = 22, 3.6%), retired with partial disability (*n* = 2, 0.3%), in school (*n* = 1, 0.2%), or had another status (*n* = 5, 0.9%). Two patients refused to provide the information. Some patients had retired with partial (*n* = 10, 1.7%) or complete disability (*n* = 17, 2.9%), had lost their employment (*n* = 9, 1.5%), were on sick leave (*n* = 129, 22%), or were working part-time (*n* = 12, 2%), all as a result of the disease’s onset.

### Subjective financial toxicity

**Fig. 1:**
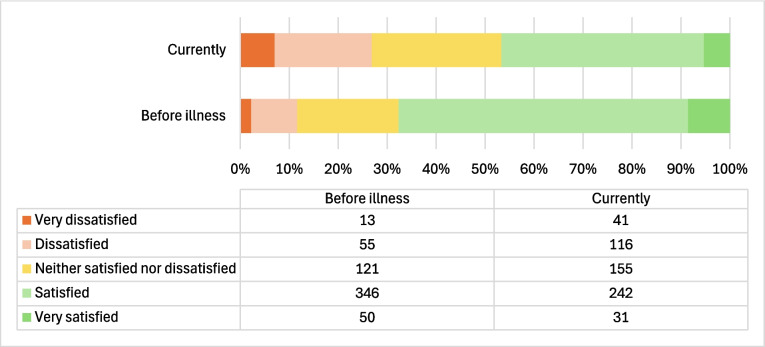
Number of patients reporting subjective change in financial capability: pre-disease versus post-disease

Figure 1 shows patients’ satisfaction with their own finances before the disease compared to their current satisfaction. One third of patients (*n* = 198, 33.7%) reported a change in satisfaction with their finances after the diagnosis, and 178 (89.9%) of them reported lower satisfaction. Two-thirds of participating patients (*n* = 389, 66.3%) reported no change. There was a notable increase in the number of patients who reported being very dissatisfied or dissatisfied with their current personal finances (*n* = 89, 15.1%), while there was a substantial decrease (*n* = 123, 21.0%) in the number of patients who expressed satisfaction with their present finances. Two people did not respond to this question.

### Objective financial toxicity

**Fig. 2:**
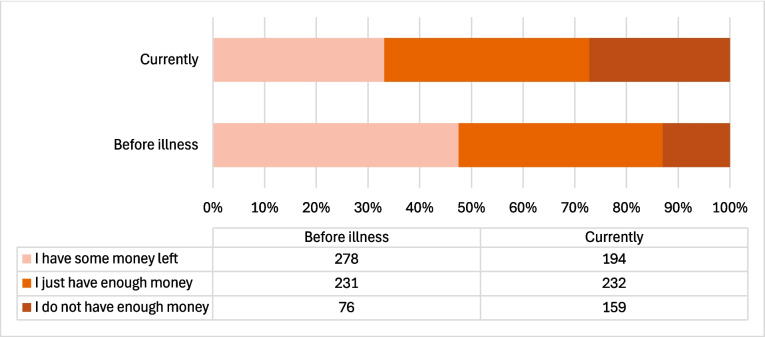
Number of patients reporting objective change in financial capability: pre-disease versus post-disease

With respect to objective FT, 166 (28.3%) patients reported a change in financial capability after diagnosis, 150 (90.4%) of them reported that it was negative. The correlation in the subjective change before and after diagnosis was statistically significant (*p* < 0.001). The majority of patients (*n* = 420, 71.6%) reported no change in financial capability (Fig. 2). Two people did not respond to this question.

The frequency of change in subjective (satisfaction with own finances) and objective (financial capability) FT and *p*-values for each of the subgroups are shown in Table 3.

**Table 3:** Frequency of change in subjective, objective FT for each of the category and *p* values

		Subjective change (*N* = 587, 100%)		*p* value		Objective change (*N* = 586, 100%)		*p* value
	*N* (%)	Increase	Decrease		*N* (%)	Increase	Decrease	
Age								
(≤ 65)	324 (55.2)	142 (43.8)	182 (56.2)	** < 0.001**	324 (55.2)	116 (35.8)	208 (64.2)	** < 0.001**
(> 65)	263 (44.8)	56 (21.3)	207 (78.7)		262 (44.8)	50 (19.1)	212 (80.1)	
Gender								
Male	291 (49.6)	81 (27.8)	210 (72.2)	**0.004**	290 (49.5)	62 (21.4)	228 (78.6)	** < 0.001**
Female	293 (49.9)	117 (39.9)	176 (60.1)		293 (50.0	104 (35.5)	189 (64.5)	
NA	3 (0.5)	-	-		3 (0.5)	-	-	
Area of living								
Urban	283 (48.2)	97 (34.3)	186 (65.7)	0.788	283	87 (30.7)	196 (69.3)	0.198
Rural	298 (50.8)	99 (33.2)	199 (66.8)		297	77 (25.9)	220 (74.1)	
NA	6 (1.0)	-	-		6	-	-	
Level of education^¥^								
1 and 2	81 (13.8)	21 (25.9)	60 (74.1)	0.223	81 (13.8)	21 (25.9)	60 (74.1)	0.852
3 and 4	320 (54.5)	115 (35.9)	205 (64.1)		319 (54.5)	89 (27.9)	230 (72.1)	
5–8	181 (30.8)	59 (32.6)	122 (64.7)		181 (30.8)	53 (29.3)	128 (70.7)	
NA	5 (0.9)				5 (0.9)	-	-	
Religion								
Religious*	406 (69.2)	134 (33.0)	272 (67.0)	0.731	406 (69.2)	122 (30.0)	284 (70.0)	0.143
Atheism	129 (22.0)	46 (35.7)	83 (64.3)		128 (22.0)	27 (21.1)	101 (78.9)	
Other	15 (2.6)	4 (26.7)	11 (73.3)		15 (2.6)	4 (26.7)	11 (73.3)	
NA	37 (6.3)	-	-		37 (6.3)	-	-	
Primary cancer diagnosis								
Breast cancer	118 (20.1)	53 (44.9)	65 (55.1)	**0.008**	118 (20.1)	40 (33.9)	78 (66.1)	**0.020**
Prostate cancer	111 (18.9)	29 (26.1)	82 (73.9)		111 (18.9)	20 (18.2)	90 (81.8)	
Lung cancer	97 (16.5)	25 (25.8)	72 (74.2)		97 (16.5)	25 (25.8)	72 (74.2)	
Head and neck cancer	66 (11.2)	19 (28.8)	47 (71.2)		66 (11.2)	15 (22.7)	51 (77.3)	
Other**	195 (33.2)	72 (36.9)	123 (63.1)		195 (33.2)	66 (33.8)	129 (66.2)	
Recurrence								
First diagnosis	494 (84.2)	158 (32.0)	336 (68.0)	**0.039**	493 (84.1)	137 (27.8)	356 (72.2)	0.506
Recurrence	93 (15.8)	40 (43.0)	53 (57.0)		93 (15.9)	29 (31.2)	64 (68.8)	
Treatment activity								
On active treatment (any)	406 (69.2)	142 (35.0)	264 (65.0)	0.297	405 (69.1)	117 (28.9)	288 (71.1)	0.582
On follow-up	180 (30.7)	55 (30.6)	125 (69.4)		180 (30.7)	48 (26.7)	132 (73.3)	
NA	1 (0.2)	**-**	**-**		1 (0.2)	-	-	
Treatment characteristics								
Multimodality treatment***	420 (71.6)	151 (36.0)	269 (64.0)	0.112	419 (71.5)	120 (28.6)	299 (71.4)	0.975
Radiation therapy (only)	77 (13.1)	18 (23.4)	59 (76.6)		77 (13.1)	21 (27.3)	56 (72.7)	
Systemic therapy (only one line)	60 (10.2)	17 (28.3)	43 (71.7)		60 (10.2)	18 (30.0)	42 (70.0)	
Surgery (only)	27 (4.6)	11 (40.7)	16 (59.3)		27 (4.6)	7 (25.9)	20 (74.1)	
NA	3 (0.5)	-	-		3 (0.5)	-	-	
Type of household								
Live alone	82 (14.0)	29 (35.4)	53 (64.6)	0.736	81 (13.8)	21 (25.9)	60 (74.1)	0.606
Live with others	505 (86.0)	169 (33.5)	336 (66.5)		505 (86.2)	145 (28.7)	360 (71.3)	
Net income per person								
≥ 900	253 (43.1)	59 (23.3)	194 (76.6)	** < 0.001**	253 (43.2)	54 (21.3)	199 (78.7)	** < 0.001**
< 900	330 (56.2)	139 (42.1)	191 (57.9)		329 (56.1)	112 (34.0)	217 (66.0)	
NA	4 (0.7)	-	-		4 (0.7)	-	-	
Employment								
Retired	301 (51.3)	58 (19.3)	243 (80.7)	** < 0.001**	300 (51.2)	52 (17.3)	248 (82.7)	** < 0.001**
Employed	256 (43.6)	129 (50.4)	127 (49.6)		256 (43.7)	105 (41.0)	151 (59.0)	
Unemployed	22 (3.7)	10 (45.5)	12 (54.5)		22 (3.8)	8 (36.4)	14 (63.6)	
Other	6 (1.0)	1 (16.7)	5 (83.3)		6 (1.0)	1 (16.7)	5 (83.3)	
NA	-	**-**	**-**		2 (0.3)	-	-	
Change of employment								
Change in employment status	49 (8.3)	18 (36.7)	31 (63.3)	0.529	49 (8.4)	12 (24.5)	37 (75.5)	0.698
No change in employment status	203 (34.6)	65 (32.0)	138 (68.0)		202 (34.5)	55 (27.2)	147 (72.8)	
Other, retired or NA	335 (57.1)	-	-		335 (57.2)	**-**	**-**	

*N *number,* NA *not available

^¥^Education levels: 1 = Incomplete primary education, 2 = Primary education, 3 = Lower or secondary vocational education, 4 = Secondary vocational, general education, 5 = Higher education, post-secondary education, 6 = Higher vocational education, 7 = University higher education qualification, 8 = Specialization, Master of Science, Doctorate

*Any religion

**Other includes: Colorectal cancer, Endometrial cancer, Lymphoma, Skin cancer (including melanoma), Esophageal and stomach cancer, Kidney cancer and Bladder cancer

***Multimodality treatment includes at least two modalities, surgery and/or radiotherapy and/or at least one line of systemic treatment

### Out-of-pocket expenses

Most patients (*n* = 500, 85%) had no major out-of-pocket expenses due to their illness and treatment. However, nearly 50% of the patients reported using nutritional supplements, about a third used alternative treatments and just under a third took OVC medicines, with the majority of patients spending 200 EUR or less on such supplements or services. The amount of out-of-pocket expenses (except for travel) that patients incurred for the most common additional financial expenses was categorized into 5 price categories, the results of which are shown in Supplementary Information 3.

**Fig. 3:**
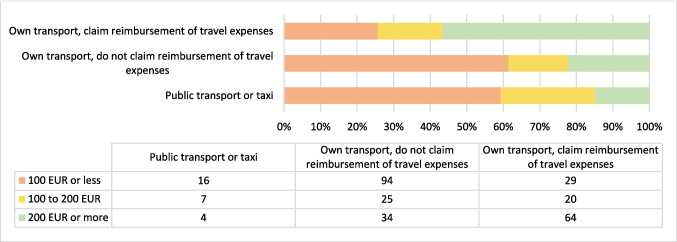
The distribution of transport costs, based on those patients who provided a cost figure (*n* = 293)

When it came to travel expenditures, all but one patient responded to this question. Most times (*n* = 241, 41.1%) patients used their own transportation to the cancer center during active treatment and did not submit a claim for reimbursement; 135 (23.0%) patients used their own transportation and submitted a claim for reimbursement of travel expenses. One quarter of patients (*n* = 143, 24.4%) used non-emergency or emergency medical transport covered by compulsory health insurance. Sixty-seven (11.4%) patients took public transportation or a taxi. The median monthly travel cost for patients during the period of most active treatment was 120 EUR (range, 2–2000 EUR). The distribution of travel costs is shown in Fig. 3.

Of the 586 patients, who responded to the question about having other persons accompany them to the medical facility, 229 (39%) were accompanied by a relative/caregiver’s free time or vacation; and 42 (7.2%) patients were accompanied by a relative/caregiver utilizing sick leave absence. About a half of patients (*n* = 315, 53.7%) usually visited the cancer center alone. One third of participating patients (*n* = 175, 29.8%) reported that at least one family member had taken at least one day off, for 83 (14%) patients their family members had taken at least one sick day, and 11 (2%) patients had at least one family member working part-time due to their illness.

## Discussion

In this study, we present the analysis of the extent of FT in patients with cancer in Slovenia, measured with a questionnaire adapted for a country with a public healthcare system. Around 25% of analyzed patients with cancer in Slovenia suffer from FT.

During the study, most patients in Slovenia had both compulsory and complementary health insurance, which meant that they did not have to pay significant costs for treatments covered by the health benefit basket. In Slovenia, insured people can retire at the age of 60 if they have completed 40 years of non-contributory pensionable service and at the age of 65 if they have completed 15 years of contributory service [24]. We assumed that a large proportion of the population will retire by the age of 65, leading to a significant change in both income and lifestyle. There was a statistically significant difference in satisfaction with own finances and financial capability between the two groups. Younger people reported changes after the disease and therefore had higher FT, which is consistent with many studies from the USA, other European countries, and Japan [25–27]. The working population tends to have higher monthly costs due to a more active lifestyle and childcare. Also, younger people tend to have less savings and are more aware of alternative treatments, so they use them more often which increases their financial burden. Contrary, retired patients do not lose income because of their illness; their pension remains the same after the diagnosis [28].

Comparable proportions of men and women were included. There was a statistically significant change in satisfaction with own finances and financial capability between the genders. More women than men reported changes after the onset of the disease. Our results are consistent with studies from the USA and Italy, both of which have confirmed higher FT in female patients [25, 29, 30]. This could be due to women taking on family and domestic responsibilities and still earning less on average than men. In European Union, women earn almost 13% less per hour than men [31].

There were no statistically significant differences observed in satisfaction with one’s personal finances or financial competence across different areas of living, religions, treatment activity, and treatment characteristics. Between patients who had been diagnosed with cancer for the first time versus patients with tumor recurrence, there was a statistically significant difference only in the change of satisfaction with their own finances.

The primary cancer diagnosis was divided into five different groups and a statistically significant difference was found between them in the change both in satisfaction with own finances and in financial capability. The group with other cancers and the group with breast cancer experienced the most change, while the group with head and neck cancer experienced the least change. On the other hand, Smith et al. found that high FT was very common in patients with head and neck cancer [11]. The differences are probably due to the unbalanced grouping of cancer types in the individual studies. In our study, among patients with breast cancer, respondents were predominantly female, which may have contributed to higher FT for the reasons mentioned above.

There was also no significant difference in change in subjective or objective FT assessment with different levels of education. We assumed that patients with higher education would have less FT. Studies in Germany and the Netherlands have both shown that higher education is associated with less FT [32, 33]. This may be because patients with higher levels of education return to work more quickly after diagnosis, resulting in less loss of income [33].

Furthermore, according to Zafar et al., larger household size should be associated with higher FT [34]. Nevertheless, our study revealed no statistically significant disparity in contentment with personal finances or financial capacity across all household sizes. Household income can vary based on employment status (e.g., sick leave, full or part-time employment, retirement), making direct comparisons between studies challenging. An important determinant of FT is also the amount of monthly income per family member. Studies in countries with different healthcare systems have confirmed that lower income per household is associated with higher financial burden [35, 36]. Our study shows a statistically significant difference in the change of satisfaction and financial capability between the income groups. Patients with lowest income were more likely to report change. The lowest income was associated with the highest FT, while the highest income was associated with the lowest FT. We concluded that patients with lower income were significantly more affected by the additional out-of-pockets expenditures due to disease and treatment.

A statistically significant difference between employment categories was found in the change in satisfaction with their own finances and financial capability. Employed patients were more likely to report a change than retired patients. We concluded that the highest level of FT was among employed patients, since they had the highest loss of income due to job loss, working part-time, retiring on partial or total disability. Unemployed patients may experience less FT due to their eligibility for social assistance [37]. Similar results were found in a study in Ireland. Employed patients had more dependents, more loans, mortgages, and less stable income. Pensioners, on the other hand, kept their pension regardless of their diagnosis [38]. We investigated whether the employment status of each employee had changed. Between the two groups (reported change versus no reported change), there were no statistically significant variations in FT experiences.

Slovenian health insurance also covers transport expenditures [35]. Many patients who travelled with their own transport and did not claim travel charges had monthly travel expenditure during the most active treatment of 100 EUR or less. Those who travelled with their own transport and claimed travel expenses were more likely to spend above 200 EUR. Patients may have been unaware of the right to claim travel expenses or the claiming process, which may be difficult especially for older patients. Around 50% of patients reported paying out-of-pocket expenditures, either in part or in full. However, higher out-of-pocket spending by patients was associated with a higher likelihood of using complementary and alternative therapies—which are not included in the conventional medical framework.

Most patients were diagnosed in the public healthcare system, mainly because of clinical symptoms and signs, with a smaller proportion diagnosed by organized screening or preventive examinations. We assume that Slovenia has a well-developed public health network, as only a small proportion of patients used self-pay screening to reach a diagnosis, as the main shortcoming of the Slovenian health system is long waiting time for some diagnostic procedures. Our findings are comparable with those from Japan, Italy, and Germany, countries with well-developed public health systems [27, 39, 40].

The strength of the survey lies in the large sample of patients with cancer. Using an extensive range of variables that were included in the questionnaire, we were able to conduct an in-depth investigation of the socioeconomic condition and financial capacity, perceived financial strain, and out-of-pocket expenses, of those who responded. Although the questionnaire was pilot-tested and overly complicated questions were simplified during the questionnaire development process, one of the main drawbacks of the survey is its length; we were faced with incomplete survey completion. In the future, it would be useful to have separate questionnaires for each diagnosis to reduce the number of questions, and it could also be shortened at the expense of the medical and demographic information available in the patients’ chart.

Our findings shed light on crucial socioeconomic and demographic disparities that contribute to FT among patients with cancer in Slovenia. Recognizing these patterns provides a valuable opportunity to address and alleviate the burden on the most affected groups. Some practical implications could be to design programs specifically aimed at younger patients, women, and those with lower income or employment challenges; establish financial advisory services integrated within healthcare facilities to guide patients on navigating treatment costs and available financial aid; and could also include psychological support for those experiencing subjective FT due to disease recurrence.

## Conclusion

We found that younger patients, women, patients with breast and other cancers (colorectal, endometrial, lymphoma, skin, esophageal, stomach, kidney, bladder, melanoma), lower income, and employment status were statistically significantly associated with the highest FT among analyzed Slovenian patients with cancer. FT was unaffected by location, education, religion, treatment activity, household type, and employment change. Disease recurrence affected only subjective FT. Most patients had both compulsory and complementary health insurance and were diagnosed through the public healthcare system. FT in Slovenia, similar to other countries with a developed public health and social care system, is significantly lower than in countries with private healthcare. Nevertheless, there are some groups of patients who are particularly vulnerable and more affected by health-care associated FT.

## Supplementary Information

Below is the link to the electronic supplementary material.

Supplementary file1 (DOCX 70 KB)

Supplementary file2 (DOCX 59 KB)

Supplementary file3 (DOCX 22 KB)

## Data Availability

The data that support the findings of this study are available on request from the corresponding author.
