# Supplementary material for: Assessment of financial toxicity in patients with cancer in Slovenia
Source: Support Care Cancer. 2025 May 30;33(6):515. doi: 10.1007/s00520-025-09591-7 (PMC12125031; doi:10.1007/s00520-025-09591-7)
Supplement: Supplementary file 1 — Supplementary file1 (DOCX 70 KB) [file 520_2025_9591_MOESM1_ESM.docx]

**Supplementary infromation 1 – Questionnaire development**

Supportive Care in Cancer

**Assessment of Financial Toxicity in Patients with Cancer in Slovenia**

Katja Vöröš^1^, Marjeta Skubic^1^, Mojca Bavdaž^2^, Petra Došenović Bonča^2^, Andraž Perhavec^1,3^, Tjaša

Redek^2^, Helena Barbara Zobec Logar^1,4^, Ivica Ratoša^1,4,*^

^1^Faculty of Medicine, University of Ljubljana, Ljubljana, Slovenia

^2^School of Economics and Business, University of Ljubljana, Ljubljana, Slovenia

^3^Division of Surgical Oncology, Institute of Oncology Ljubljana, Ljubljana, Slovenia

^4^Division of Radiotherapy, Institute of Oncology Ljubljana, Ljubljana, Slovenia

**Search method**

We conducted a comprehensive literature search using PubMed, Google Scholar, and Web of Science to identify peer-reviewed original research articles, meta-analyses, and reviews published until February 2024. The inclusion criteria encompassed studies involving humans, articles published in English. Our search employed the following terms, as shown in **Figure A1**. Additionally, we manually examined the bibliographies of the reviewed articles to broaden the scope of the literature search. **Figure A2** illustrates the PRISMA flowchart.

| **Cancer [Title/Abstract]** | AND | **Financial toxicity [Title/Abstract]** | AND | **Quality of life, instruments [Title/Abstract]** | AND | **Survivorship [Title/Abstract]** |
| --- | --- | --- | --- | --- | --- | --- |
|  |  |  |  |  |  |  |
| **Keywords 1** |  | **Keywords 2** |  | **Keywords 3** |  | **Keywords 4** |
| OR |  | OR |  | OR |  | OR |
| ***Neoplasm[Mesh]*** |  | ***Financial stress[Mesh]*** |  | ***Quality of Life[Mesh]*** |  | ***Survivors[Mesh]*** |
| Cancer |  | Financial toxicity* |  | Life quality |  | Survivor* |
| Tumor |  | Financial challenge* |  | Health related quality of life |  | Survive* |
| Tumour |  | Financial pressure* |  | QOL |  |  |
| Malignan* |  | Financial hardship* |  | HRQOL |  |  |
| Neoplasm* |  | Financial burden* |  | Wellbeing |  |  |
| Neoplasia |  | Financial distress* |  | Questionnaire |  |  |
| Neoplastic |  | Financial impact* |  | COST-FACIT |  |  |
| Carcinoma |  | Financial cost* |  |  |  |  |
| Oncolog* |  | Financial wellbeing |  |  |  |  |
|  |  | Economic toxicit* |  |  |  |  |
|  |  | Economic challenge* |  |  |  |  |
|  |  | Economic pressure* |  |  |  |  |
|  |  | Economic hardship* |  |  |  |  |
|  |  | Economic burden* |  |  |  |  |
|  |  | Economic distress* |  |  |  |  |
|  |  | Economic impact* |  |  |  |  |
|  |  | Economic cost* |  |  |  |  |
|  |  | Economic wellbeing |  |  |  |  |

**Figure A1**: Search terms.

**Figure A2**: PRISMA flow diagram.

Records after duplicates removed
(n = 165)

Full-text articles assessed for eligibility (n = 151)

Studies included in qualitative synthesis
(n = 151)

Records screened
(n = 165)

Records excluded
(n = 10)

Full-text articles excluded, with reasons (n = 0)

## Included

## Eligibility

## Screening

## Identification

Additional records identified through other sources
(n = 22)

Records identified through database searching
(n = 230)

**Questionnaire development**

During the pilot testing phase, we conducted one-on-one interviews with 50 patients to complete the questionnaire. The two interviewers were always present to provide more explanations for particular questions, write down the patients' notes, evaluate their comprehension, and make suggestions for improvement. In the next phase, we began evaluating an independently created questionnaire; **Figure A3** depicts the questionnaire creation process.

Personal interview

Joint peer review and adjustment

Final version

**Figure A3: The process of developing an independently developed questionnaire.**

After 50 interviews, an expert review was conducted with the assistance of experts in oncology, methodology, statistics, and economics. The questionnaire was then rearranged and simplified, as illustrated in **Figure A4**.

45 main questions

38 main questions

33 main questions

5 sub-questions

**Figure A4: The process of redesigning the questionnaire based on peer review.**

Similar questions were eliminated, merged, or reorganized; overly complex questions were simplified, and supplementary explanations were provided. Seven questions were eliminated from the original set of 45, resulting in a total of 38 questions, which were categorized into 33 main questions and 5 sub-questions.

Initially, multiple potential responses existed regarding the question associated with the specified diagnosis.

Patients with multiple cancer diagnoses were uncertain about the appropriate labeling of their conditions. The question was modified to emphasize solely the current diagnosis, with all subsequent inquiries pertaining to this diagnosis. Issues also emerged regarding the timing of diagnosis. In the initial testing phase, we requested only the year of diagnosis. However, following expert review and analysis of patient documentation, it became evident that incorporating the month of diagnosis is beneficial. This is due to the significant time variation within a year, which leads to considerable differences in costs for patients diagnosed with cancer in the same year.

When inquired about the number of individuals residing in a joint household, respondents frequently excluded themselves from consideration. Therefore, we opted to emphasize the phrase that includes "with you." In addressing whether the respondents contributed the most income, we initially provided only two options: "yes" or "no." Following feedback, we recognized the need to include an additional option to account for those who contribute equally to the joint household, and we made the necessary correction. The patients perceived the inquiry regarding health insurance as overly complex; therefore, we included clarifications in parentheses detailing the coverage of individual health insurance plans.

The finalized questionnaire comprised four primary sections: illness and treatment, socioeconomic status, targeted inquiries regarding financial toxicity, and sociodemographic information. Two additional questions pertaining to the subjective evaluation of treatment costs were whether patients would like to know the cost of their care or if they might already be aware of it. The questionnaire's individual sections with content are presented in **Table A1**.

| **ILLNESS AND TREATMENT** | **TARGETED INQUIRIES REGARDING FINANCIAL TOXICITY** |
| --- | --- |
| Current cancer diagnosis | Evaluation of financial capacity prior to illness |
| Time of diagnosis | Evaluation of present financial capacity |
| First or second diagnosis of the same cancer | Financial considerations prior to health issues |
| A method for cancer detection | Current Financial Status |
| Treatment activity | Employment Status Prior to Illness |
| Type of treatment | Post-illness employment status |
| **SOCIOECONOMIC STATUS** | Utilization of patient sick leave |
| Household type | Modes of transportation to reach the hospital |
| Number of people living in a joint household | Accompanying persons en route to the hospital |
| Regularly employed persons in a joint household | Effects on the working hours of family members |
| Maximum income contribution before diagnosis | Additional costs and their respective amounts |
| Maximum income contribution after diagnosis | Assessment of the expenses associated with formal treatment |
| Type of health insurance |  |
| Net monthly income per family member |  |
| **SOCIODEMOGRAPHIC INFORMATION** | |
| Gender | Type of settlement |
| Age | Education |
| Region of residence | Religion |

**Table A1.: The content of the questionnaire, organized into individual sections.**
