# Supplementary material for: Assessment of financial toxicity in patients with cancer in Slovenia
Source: Support Care Cancer. 2025 May 30;33(6):515. doi: 10.1007/s00520-025-09591-7 (PMC12125031; doi:10.1007/s00520-025-09591-7)
Supplement: Supplementary file 2 — Supplementary file2 (DOCX 59 KB) [file 520_2025_9591_MOESM2_ESM.docx]

**Supplementary information 2 - Questionnaire**

Supportive Care in Cancer

**Assessment of Financial Toxicity in Patients with Cancer in Slovenia**

Katja Vöröš^1^, Marjeta Skubic^1^, Mojca Bavdaž^2^, Petra Došenović Bonča^2^, Andraž Perhavec^1,3^, Tjaša

Redek^2^, Helena Barbara Zobec Logar^1,4^, Ivica Ratoša^1,4,*^

^1^Faculty of Medicine, University of Ljubljana, Ljubljana, Slovenia

^2^School of Economics and Business, University of Ljubljana, Ljubljana, Slovenia

^3^Division of Surgical Oncology, Institute of Oncology Ljubljana, Ljubljana, Slovenia

^4^Division of Radiotherapy, Institute of Oncology Ljubljana, Ljubljana, Slovenia

1. **DISEASE AND TREATMENT**
2. Indicate your current diagnosis.

- Breast cancer
- Lung cancer
- Colorectal cancer
- Prostate cancer
- Skin cancer, including skin melanoma
- Oesophageal or gastric cancer
- Urinary bladder cancer
- Lymphoma
- Kidney cancer
- Uterine cancer
- Leukaemia
- Testicular cancer
- Head and neck cancer
- Other: ________________________________________________________

1. Write down the year and month when you were first diagnosed with this type of cancer (same diagnosis as in question 1).
2. Was it the first time you were diagnosed with this type of cancer?
   - Yes
   - No, the cancer recurred less than a year after the first diagnosis
   - No, the cancer recurred more than a year after the first diagnosis
3. How was your cancer first detected? Multiple answers are possible.

- Based on clinical symptoms and signs confirmed by a self-pay examination
- Based on clinical symptoms and signs confirmed under public health care (with a health insurance card)
- Through an organised screening programme (DORA, ZORA, Svit)
- Preventive or routine check (e.g. every five years, managerial, systematic health check at work)
- Self-pay (e.g. examination required for obtaining a driver’s licence)
- Other: _________________________________________________________

1. If you had a self-pay examination, state the reason for it. Multiple answers are possible.
   - Waiting times in public healthcare are too long
   - Private healthcare offers superior quality
   - Poor communication with my doctor (I was unable to make an appointment with my personal physician)
   - It’s hard to get hold of my doctor
   - I do not have a personal physician
   - Other: ________________________________________________________
2. If you had a self-pay examination, indicate what medical service you received.

_______________________________________________________________________________

Is your cancer currently being actively treated?

- - YES, active treatment of the disease is currently underway (select this option even if active treatment has been temporarily stopped but will continue (e.g. breaks between chemotherapies))
  - NO, but the current approach is "watch and wait"
  - NO, active treatment is complete (cancer cured)
  - NO, palliative care only

1. Indicate the course of your cancer treatment. Multiple answers are possible.
   - Surgical treatment
   - Radiation
   - Hormone therapy
   - Chemotherapy
   - Targeted therapy/immunotherapy
   - Other: _______________________________________________________
2. **SOCIO-ECONOMIC SITUATION**
3. What kind of household do you live in?
   - I live alone
   - I live with one or several other people/family members
   - I live alone and with home care
   - I live in a nursing home
   - Other: ________________________________________________________

7. 1 If you answered in the previous question that you live in a nursing home or with home care, please indicate whether cancer is the reason for that.

- - Yes
  - No

1. Write down the number of people living in your household, including yourself.
2. Write down the number of people in your household who are currently in full-time employment, including yourself.
3. Are you the one who currently contributes the most income?
   - Yes
   - No
   - Other: ________________________________________________________
4. Are you the person who contributed the most income before your cancer diagnosis?
   - Yes
   - No
   - Other: ________________________________________________________
5. Which health insurance do you have? Multiple answers are possible.
   - Compulsory health insurance (I have a ZZZS health insurance card)
   - Supplementary health insurance to cover co-payments (covers the difference up to the full price of health services, e.g. specialist examinations, diagnostics, surgeries)
   - Additional health insurance (e.g. for premium services, faster access to specialists, etc.)
   - I do not have compulsory health insurance
   - Other: ________________________________________________________

Indicate the income per family member in your household. Add up all your monthly net incomes and divide by the number of people living in the same household as you.

Up to €300

From €300 to €600

From €600 to €900

From €900 to €1200

From €1200 to €1500

More than €1500

1. **FOCUSED QUESTIONS ON FINANCIAL CAPACITY**
2. How would you have assessed your financial situation before your illness?

| Very dissatisfied | Dissatisfied | Neither satisfied nor dissatisfied | Satisfied | Very satisfied |
| --- | --- | --- | --- | --- |
|  |  |  |  |  |

1. How would you rate your current financial situation?

| Very dissatisfied | Dissatisfied | Neither satisfied nor dissatisfied | Satisfied | Very satisfied |
| --- | --- | --- | --- | --- |
|  |  |  |  |  |

1. Before the onset of your illness, what was your financial situation at the end of the month?

- I had some money left
- I just had enough money
- Not enough money

1. What is your current financial situation at the end of the month?

- I have some money left
- I just have enough money
- Not enough money

Indicate your employment status before your illness.

- - On sick leave
  - Full-time employed, self-employed
  - Part-time employed, self-employed
  - Unemployed, actively seeking employment
  - Unemployed, unable/unwilling to look for a job due to illness
  - Retired with partial disability
  - Retired with full disability
  - Retired
  - Still getting my education
  - Other: ________________________________________________________

1. Was the cancer mentioned at the beginning of the questionnaire the reason for the change in employment status?
   - No
   - Yes, I'm on sick leave
   - Yes, full-time employed, self-employed
   - Yes, part-time employed, self-employed
   - Yes, unemployed, actively seeking employment
   - Yes, unemployed, unable/unwilling to look for a job due to illness
   - Yes, retired with partial disability
   - Yes, retired with full disability
   - Yes, I changed jobs for a lower paid job than before diagnosis
   - Still getting my education
   - Other: ________________________________________________________
2. Have you ever been temporarily absent from work because of cancer and used your right to compensation during the temporary absence ("been on sick leave")?

- Yes
- No

20. 1 If you answered yes to the previous question, write down the total amount of time (days/weeks/months) you were absent from work and for which you used your right to wage compensation due to temporary absence from work ("sick leave").

1. What type of transport do you use most often when you come to the Institute of Oncology Ljubljana because of your cancer?
   - Public transport or a taxi
   - Own transport, I do not claim reimbursement of travel expenses
   - Own transport, I claim reimbursement of travel expenses
   - Non-emergency or other medical transport covered by health insurance
2. 1 How much are or were your monthly transport costs at the time of your most active treatment? Include parking fees as well.

€ ________

1. Is there a person accompanying you on your way to the medical facility?
   - Yes, a person using their own free time
   - Yes, the accompanying person is an active worker and uses sick leave to accompany me
   - Yes, the accompanying person is an active worker and uses their vacation time
   - No
2. How has your cancer affected the working hours of other working family members? Multiple answers are possible.
   - At least one member of your family took at least one day off work
   - At least one member of your family has taken sick leave at least once
   - At least one member of my family works part-time because of my disease
   - The diagnosis did not affect the working hours of the other full-time family members
3. Have there been other major expenses that you have paid for yourself as a result of your cancer that have increased your financial burden (e.g. renovation of your home, a bigger car because of a wheelchair)?
   - Yes
   - No
4. Indicate the amount of expenses you or your family members have paid for so far during the entire period of your illness.

|  | I fully paid for it myself | I partly paid for it (insurance, donations, etc.) | Partly covered by compulsory or supplementary insurance (ZZZS, ZPIZ) | Fully covered by compulsory or supplementary insurance (ZZZS, ZPIZ) | I need it, but I cannot afford it | I do not need/use it | Amount of own personal expenses (in euros) |
| --- | --- | --- | --- | --- | --- | --- | --- |
| Self-pay examinations |  |  |  |  |  |  |  |
| Wheelchair |  |  |  |  |  |  |  |
| Adaptations for living at home (nursing bed and other equipment, adaptations to bathroom and living areas, house lift, etc.) |  |  |  |  |  |  |  |
| Crutches |  |  |  |  |  |  |  |
| Orthopaedic braces |  |  |  |  |  |  |  |
| Compression stockings, belts, gloves |  |  |  |  |  |  |  |
| Wigs |  |  |  |  |  |  |  |
| Breast prostheses |  |  |  |  |  |  |  |
| Car adaptations |  |  |  |  |  |  |  |
| Help with personal and medical care |  |  |  |  |  |  |  |
| Other help, not related to nursing (cooking, cleaning, gardening, etc.) |  |  |  |  |  |  |  |
| Over-the-counter medicines |  |  |  |  |  |  |  |
| Food supplements (vitamins, minerals, Prosure, Ensure, etc.) |  |  |  |  |  |  |  |
| Physiotherapy, massage, lymphatic drainage |  |  |  |  |  |  |  |
| Psychological/psychiatric  treatment |  |  |  |  |  |  |  |
| Cannabis preparations and other cannabinoids (resin, buds, suppositories, CBD drops) |  |  |  |  |  |  |  |
| Bioresonance |  |  |  |  |  |  |  |
| Healers |  |  |  |  |  |  |  |
| Meditation and relaxation techniques |  |  |  |  |  |  |  |
| Other (write down): |  |  |  |  |  |  |  |

1. How much do you think your official treatment covered by compulsory insurance costs (how much does it cost to see a doctor, undergo treatment, take medication, have check-ups, scans, etc.)? Try to estimate the average monthly cost during your most active treatment period.
2. Would you like to know how much your treatment costs?
   - Yes
   - No
3. **SOCIO-DEMOGRAPHIC DATA**
4. Indicate your gender.

- Male
- Female
- I do not wish to answer
- Other

1. State your age.
2. Which region do you live in?
   - Mura
   - Drava
   - Carinthia
   - Savinja
   - Central Sava
   - Lower Sava
   - Southeast Slovenia
   - Central Slovenia
   - Upper Carniola
   - Littoral–Inner Carniola
   - Gorizia
   - Coastal–Karst
   - Other: ___________________________________________________________
3. How would you describe the area where you live?

Urban

- Rural

1. What is your highest level of formal education?

1 – Incomplete primary education

2 – Primary education

3 – Lower or secondary vocational education

4 – Secondary vocational, general education

5 – Higher education, post-secondary education

6 – Higher vocational education (includes first-cycle Bologna studies)

7 – University higher education qualification (includes second-cycle Bologna studies)

8 – Specialisation, Master of Science, Doctorate

1. Indicate your religion.

- Christianity
- Judaism
- Islam
- Atheism
  - Other: ____________________________________________________________
