# Supplementary material for: Assessment of financial toxicity in patients with cancer in Slovenia
Source: Support Care Cancer. 2025 May 30;33(6):515. doi: 10.1007/s00520-025-09591-7 (PMC12125031; doi:10.1007/s00520-025-09591-7)
Supplement: Supplementary file 3 — Supplementary file3 (DOCX 22 KB) [file 520_2025_9591_MOESM3_ESM.docx]

**Online resource 3**

Supportive Care in Cancer

**Assessment of Financial Toxicity in Patients with Cancer in Slovenia**

Katja Vöröš^1^, Marjeta Skubic^1^, Mojca Bavdaž^2^, Petra Došenović Bonča^2^, Andraž Perhavec^1,3^, Tjaša

Redek^2^, Helena Barbara Zobec Logar^1,4^, Ivica Ratoša^1,4,*^

^1^Faculty of Medicine, University of Ljubljana, Ljubljana, Slovenia

^2^School of Economics and Business, University of Ljubljana, Ljubljana, Slovenia

^3^Division of Surgical Oncology, Institute of Oncology Ljubljana, Ljubljana, Slovenia

^4^Division of Radiotherapy, Institute of Oncology Ljubljana, Ljubljana, Slovenia

**Supplementary Figure C1: Distribution of all out-of-pocket expenses (except for travel expenditures).**

| **OUT-OF-POCKET-EXPENSES** | **NUMBER** | **PERCENTAGE (%)** |
| --- | --- | --- |
| Dietary supplements | 276 | 47.0 |
| Alternative treatments | 184 | 31.3 |
| Over the counter medicines | 178 | 30.3 |
| Self-pay check-ups | 117 | 19.9 |
| Physiotherapy | 99 | 16.9 |
| Wig | 88 | 15.0 |
| Psychological treatment or meditation | 85 | 14.5 |
| Non-nursing care | 37 | 6.3 |
| Compression stockings | 34 | 5.8 |
| Home adaptations | 31 | 5.3 |
| Breast prostheses | 27 | 4.6 |
| Personal and care aids | 27 | 4.6 |
| Crutches | 23 | 3.9 |
| Orthopaedic corsets | 14 | 2.4 |
| Wheelchairs | 12 | 2.0 |
| Vehicle adaptations | 8 | 1.4 |
| Other | 19 | 3.2 |

**Supplementary Table C1: Details on out-of-pocket expenses.**
